# A thinner endometrium is associated with a greater risk of preterm delivery after fresh cleavage embryo transfer but not after blastocyst transfer: a retrospective cohort study of 11,111 singleton live births

**DOI:** 10.3389/fendo.2025.1574123

**Published:** 2025-05-30

**Authors:** Tian Ye, Huijuan Kong, Zhiqin Bu, Wenqian Fan, Linqing Du, Jing Li, Yihong Guo

**Affiliations:** ^1^ Centre for Reproductive Medicine, The First Affiliated Hospital of Zhengzhou University, Zhengzhou, China; ^2^ Henan Key Laboratory of Reproduction and Genetics, The First Affiliated Hospital of Zhengzhou University, Zhengzhou, China; ^3^ Henan Provincial Obstetrical and Gynecological Diseases (Reproductive Medicine) Clinical Research Center, The First Affiliated Hospital of Zhengzhou University, Zhengzhou, China; ^4^ Henan Engineering Laboratory of Preimplantation Genetic Diagnosis and Screening, The First Affiliated Hospital of Zhengzhou University, Zhengzhou, China

**Keywords:** endometrial thickness, preterm delivery, cleavage-stage embryo transfer, blastocyst transfer, hCG trigger day

## Abstract

**Background:**

Endometrial thickness (EMT) has been confirmed to be associated with pregnancy outcomes after *in vitro* fertilization/intracytoplasmic sperm injection-embryo transfer (IVF/ICSI-ET), but studies on its relationship with neonatal outcomes are still limited. To our knowledge, this study is the first to investigate the relationship between EMT on the day of hCG trigger and the risk of preterm delivery (PTD) in populations undergoing cleavage-stage embryo transfer and blastocyst transfer, respectively.

**Methods:**

This study was a retrospective cohort study that included singleton live birth cycles of women who underwent autologous IVF/ICSI-ET at the Reproductive Medicine Center of the First Affiliated Hospital of Zhengzhou University from January 2016 to December 2023. The main study outcome was PTD. The relationship between EMT and PTD was explored using logistic regression in different models. These models were adjusted for baseline characteristics, cycle treatment parameters and maternal pregnancy complications among populations undergoing cleavage-stage embryo and blastocyst transfer.

**Results:**

In both the unadjusted model and Model I, which was adjusted for baseline characteristics, compared with that in the EMT 7.5–12 mm group, the risk of PTD was significantly greater in the EMT < 7.5 mm group and significantly lower in the EMT ≥ 12 mm group (*P* < 0.05). In Model II, which was adjusted for all potential confounding factors, including pregnancy conditions, an EMT ≥ 12 mm retained its independent protective effect against PTD in both populations. In contrast, an EMT < 7.5 mm and PTD (OR 2.19; 95% CI, 0.82–5.88; P = 0.118) did not significantly correlated in the blastocyst transfer population. However, in patients undergoing cleavage-stage embryo transfer, an EMT < 7.5 mm remained an independent risk factor for PTD (OR 2.14; 95% CI, 1.09–4.21; P = 0.027).

**Conclusions:**

A thin endometrium on the day of hCG trigger is independently associated with an increased risk of PTD in patients undergoing cleavage-stage embryo transfer but not in those undergoing blastocyst transfer. In contrast, a thick endometrium significantly reduces the risk of PTD in both populations.

## Introduction

Assisted reproductive technology (ART) has been widely applied globally, with a continuous increase in the number of infants born through ART (1). The safety of ART to offspring has always been a concern. ART is associated with various adverse short-term health outcomes, such as preterm delivery (PTD), low birth weight (LBW), small for gestational age (SGA), congenital malformations, and imprinting disorders (2–4). Even in reports of singleton births, similar negative outcomes have been reported (5). However, the pathogenesis of these effects is still poorly understood. Whether this risk is due to *in vitro* fertilization/intracytoplasmic sperm injection (IVF/ICSI) treatment or to the underlying maternal infertility itself remains a matter of debate (6). Therefore, exploring factors associated with adverse maternal and neonatal outcomes in ART is crucial.

Endometrial thickness (EMT) is the most commonly used indicator for assessing endometrial receptivity. The endometrium, as the innermost layer of the uterus, plays a vital role in embryo implantation and development. A thinner EMT is associated with reduced pregnancy rates (7) and an increased risk of ectopic pregnancy (8, 9), both in fresh and frozen embryo transfer (FET) cycles. Some studies suggest that an EMT above 14 mm may be associated with an increased risk of miscarriage (10). Additionally, EMT is associated with several adverse perinatal outcomes caused by placental abnormalities, such as placental abruption, placenta accreta spectrum (PAS) disorders, fetal growth restriction (FGR), and hypertension disorders of pregnancy (HDP) (11–13). In summary, EMT has been proven to be closely related to ART outcomes.

However, most current studies focus on the association between EMT and pregnancy outcomes, with limited research further exploring the relationship between thin EMT and neonatal outcomes in IVF. Moreover, a unified standard cutoff value for a thin endometrium is lacking. A recent study indicated that an EMT ≤ 7.8 mm is an independent predictor of increased PTD risk in singleton live births during fresh cycles (14). In FET cycles, an EMT ≤ 8 mm is associated with an increased risk of PTD (15). In both fresh and FET cycles, an EMT < 8 mm is considered related to lower average birth weights (12, 16). Some studies suggest that in fresh cycles, an EMT ≤ 10 mm is associated with an increased risk of LBW compared with women with an EMT > 12 mm (17). This association may be due to the reduced selective capacity caused by a thinner endometrium. Another study revealed that an EMT < 7.5 mm is associated with an increased risk of SGA after fresh embryo transfer (18). Compared with women with an EMT > 12 mm, women with an EMT ≤ 7.5 mm had a twofold increased risk of SGA (19). However, Huang et al. demonstrated that EMT is not an independent predictor of adverse perinatal outcomes in intrauterine insemination (IUI) cycles (20). Therefore, the EMT may not only be related to recent pregnancy outcomes but also affect the long-term development of embryos and placentas, thereby influencing the safety of offspring. However, the mechanisms by which EMT affects neonatal outcomes remain unclear. It may be related to embryo implantation and placental development. Moreover, in most previous studies, the type of embryo transfer was not restricted. In contrast, our present study explored the relationship between EMT and PTD separately in populations undergoing cleavage-stage embryo transfer and blastocyst transfer, thereby filling the gap in previous research.

Preterm delivery is the leading cause of mortality in children under 5 years of age and in newborns (21). Thus, we chose the PTD as a representative evaluation index for offspring safety. Based on previous studies, we selected 7.5 mm and 12 mm as the optimal upper and lower cutoff values for the EMT, respectively. In previous studies on EMT and neonatal outcomes, the types of embryos transferred were not distinguished (14, 15). To our knowledge, we are the first to explore the relationship between EMT and PTD in populations undergoing fresh cleavage-stage embryo transfer and blastocyst transfer.

## Materials and methods

### Study design and population

**Figure 1:**
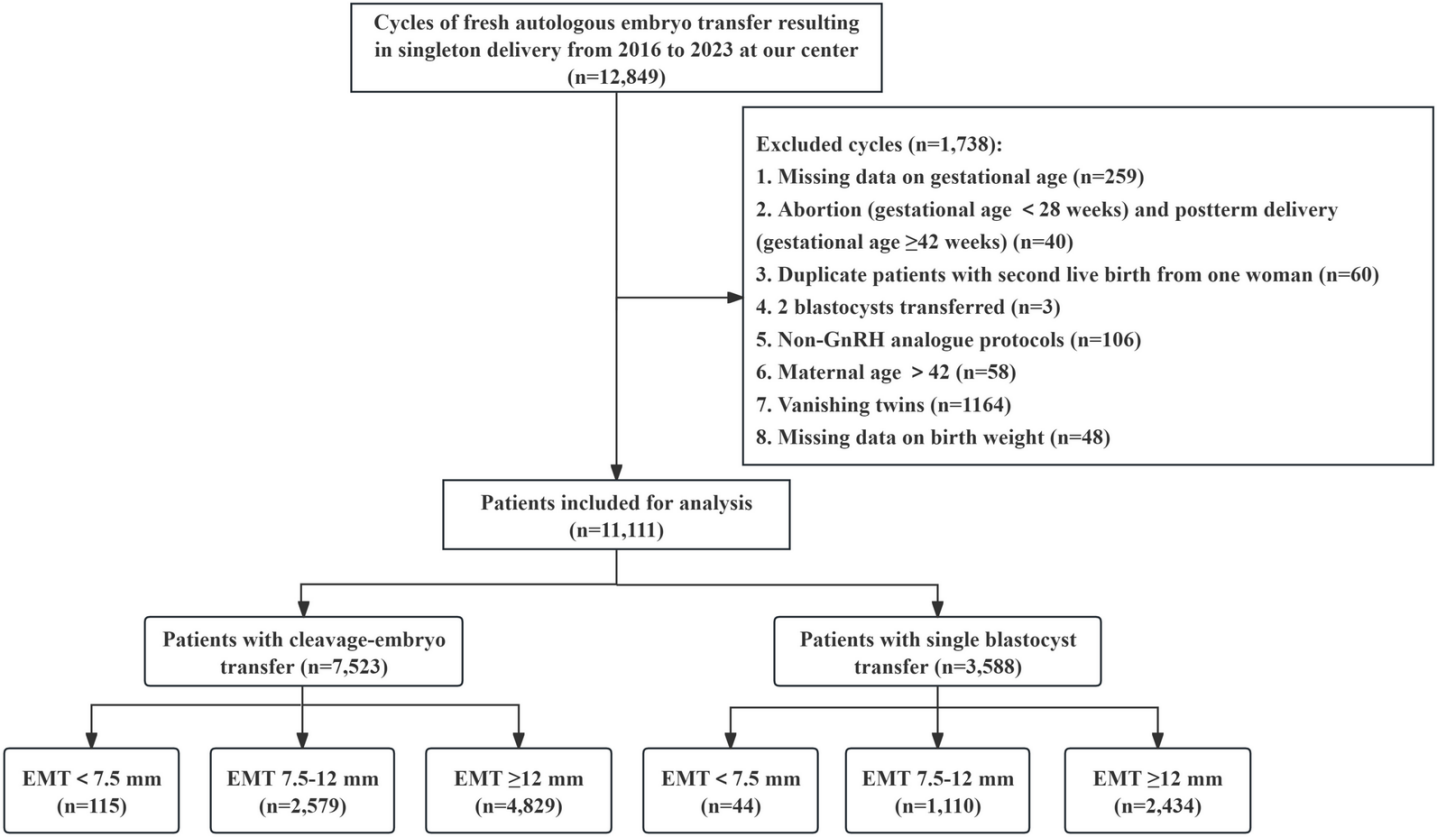
Flowchart of patient screening.

This work was a retrospective cohort study of women who underwent autologous IVF/ICSI treatment and received fresh embryo transfer at the Reproductive Medical Center of the First Affiliated Hospital of Zhengzhou University from January 2016 to December 2023, resulting in live singleton births. Our exclusion criteria included the following: (i) Patients with miscarriages (gestational age <28 weeks) and postterm delivery (gestational age ≥42 weeks); (ii) Patients who had two blastocysts transferred; (iii) Patients who did not use the GnRH analog protocol; (iv) Patients over 42 years of age; (v) Patients with vanishing twins; (vi) For patients with multiple deliveries, only their first delivery was included; and (vii) Patients with missing information on the primary outcome measures (gestational week or birthweight). We collected baseline blood pressure information for all patients. This information was included as a covariate in the multivariate regression model. Therefore, our study did not exclude patients with chronic hypertension. According to convention and clinical practice, we defined < 7.5 mm as the cutoff value for a thin endometrium (7, 18, 22). Consistent with previous studies, we chose 12 mm as the upper cutoff for a thick endometrium (11, 19, 23). All included cases were recorded and organized by dedicated nurses at our center to ensure data integrity and authenticity. A flow chart of the patient screening process is shown in **Figure 1**. Finally, a total of 11,111 patients were included in this study. This study strictly adhered to the relevant requirements of the World Medical Association’s Declaration of Helsinki and was approved by the Ethics Committee of the First Affiliated Hospital of Zhengzhou University (Ethics No. 2024-KY-0386-001). All patients signed informed consent before entering the IVF cycle for the use of their medical records for data analysis.

### Controlled ovarian stimulation protocols and EMT measurement

This study included ovarian stimulation protocols using GnRH analogs, including the early-follicular phase long-acting GnRH-a long (EFLL) protocol, luteal phase short-acting GnRH-a (LPS) protocol, and GnRH antagonist protocol. Individualized treatment was determined for each patient based on ovarian reserve, female age, and other characteristics. The procedures for the EFLL and LPS protocols are described in our previous studies (24). The GnRH antagonist protocol was initiated by administering exogenous gonadotropin (Gn) on the 2nd–4th days of menstruation. According to the personal experience of physicians, a fixed or flexible GnRH-ant protocol was performed using 0.25 mg daily of GnRH-ant (Cetrotide; Merck Serono) from Day 6 of stimulation or as soon as the diameter of the leading follicle reached 12–14 mm. When two dominant follicles reached a diameter of ≥18 mm or three follicles reached a diameter of ≥17 mm, HCG (Zhuhai Lizhu Group, Lizhu Pharmaceutical Factory) was administered to trigger ovulation. Oocytes were retrieved 36 hours after triggering.

All patients underwent EMT measurement on the day of hCG trigger. The measurement was performed by highly trained and experienced physicians in ultrasound monitoring and was recorded in millimeters. Endometrial thickness was defined as the maximum distance between the hyperechoic interface between the endometrium and myometrium in the sagittal plane, approximately 1 cm below the uterine fundus.

Fertilization was performed using conventional IVF or ICSI methods based on the quality of the male semen. The methods for handling oocytes after retrieval and embryo culture have been described in our previous studies (25). All included patients underwent cleavage embryo or blastocyst transfer on Day 3 or Day 5 after oocyte retrieval. The morphological scoring of cleavage embryos was performed by experienced embryologists based on classification systems and our practical experience, with specific criteria detailed in previously published studies (25). High-quality cleavage embryos were defined as Grade I and II, whereas Grade III and IV embryos were defined as low quality, with Grade IV embryos not considered for transfer. Blastocyst scoring was based on the Gardner scoring system (26), which evaluates the blastocyst cavity, inner cell mass (ICM) development, and trophectoderm (TE) appearance, as well as the number and structural density of trophoblasts. High-quality blastocysts were defined as AA, AB, BA, and BB, whereas BC, CB, AC, and CA blastocysts were considered of lower quality. Blastocyst-grade CCs were not considered for transfer. Only blastocysts that expanded on the morning of Day 5 after oocyte retrieval were considered for fresh transfer. Luteal phase support began after oocyte retrieval and continued until 10 weeks of pregnancy. Our center utilizes two luteal phase support regimens after fresh embryo transfer: (i) oral dydrogesterone (10 mg, three times daily) and Utrogestan vaginal soft capsules (200 mg, twice daily) and (ii) oral dydrogesterone (10 mg, three times daily) and Crinone 8% vaginal gel (90 mg, once daily). The choice of luteal phase support regimen was based on physician experience and patient preference, independent of endometrial thickness.

### Neonatal follow-up

At our center, the neonatal follow-up system is managed by well-trained nurses. The nurses responsible for neonatal follow-up were trained by archivists through the use of standardized questionnaires. Nurses conduct surveys of couples by phone at each stage of pregnancy until delivery. Basic information, including complications related to pregnancy (e.g., gestational diabetes, hypertensive disorders, placental abnormalities), gestational weeks, mode of delivery, newborn gender, date and place of birth, birth weight, Apgar scores, and neonatal diseases, was collected via a standardized interview protocol. Finally, the content of the telephone follow-ups was meticulously recorded by the nurses in our Clinical Reproductive Medicine Management System/Electronic Medical Record Cohort Database (CCRM/EMRCD).

### Outcome measures and definitions

We collected data including demographic and cycle parameters, such as maternal age, paternal age, maternal body mass index (BMI), paternal BMI, duration of infertility, gravidity, parity, miscarriage history, type of infertility, cause of infertility, cycle rank, maternal education level, paternal education level, anti-Müllerian hormone (AMH), baseline antral follicle count (AFC), baseline systolic and diastolic blood pressure, baseline follicle-stimulating hormone (bFSH), controlled ovarian stimulation (COS) protocol, total gonadotropin dosage, days of stimulation, estradiol (E2) levels on the trigger day, progesterone (P) levels on the trigger day, number of oocytes retrieved, fertilization methods, and morphological scores of transferred embryos. Additionally, we collected information on maternal pregnancy complications, including placental abnormalities such as placenta previa (PP), placental abruption, PAS, HDP, and gestational diabetes mellitus (GDM).

A live birth was defined as the delivery of any surviving infant at ≥ 28 weeks of gestation. The primary outcome of this study was PTD. Other neonatal outcomes analyzed included gestational weeks, birth weight, LBW, macrosomia, SGA, and LGA. PTD was defined as delivery occurring at a gestational age of ≥ 28 weeks and < 37 weeks. To diagnose SGA and LGA, we referred to birth weight percentiles (27). SGA and LGA were defined as birth weights < 10th and > 90th percentiles, respectively, for the same gestational age. LBW and macrosomia were identified as birthweight <2500 and ≥ 4000 g, respectively.

### Statistical analysis

All the statistical analyses were performed using R software (version 4.2.2), along with the use of MSTATA software (https://www.mstata.com/). Continuous variables were tested for normality via the Kolmogorov–Smirnov test. Nonnormally distributed data are expressed as the median (M) and interquartile range (Q25, Q75). The Kruskal–Wallis rank sum test was used to analyze differences between groups. Categorical data are expressed as frequencies and percentages. Comparisons between study groups for these measured variables were performed using Pearson’s chi-square test or Fisher’s exact probability test.

A logistic regression model was used to assess the relationship between EMT on the trigger day and PTD. Confounding factors were included in the adjusted models. Model I adjusted for the demographic baseline characteristics and cycle treatment parameters of the couples, including maternal and paternal age, maternal and paternal BMI, infertility duration, gravidity, parity, history of miscarriage, type of infertility, cause of infertility, cycle rank, maternal and paternal education level, AMH, AFC, baseline systolic and diastolic blood pressure, bFSH, COS protocol, total gonadotropin dose, days of stimulation, E2 and P levels on the trigger day, number of oocytes retrieved, fertilization method, number of embryos transferred, and morphological quality of embryos transferred. Model II was adjusted for all covariables in Model I, plus pregnancy complications and fetal intrauterine development indicators, including placental disorders, PROM, GDM, HDP, gender of the newborn, SGA, and LGA. Crude odds ratios (ORs) and adjusted odds ratios (aORs) with 95% confidence intervals (CIs) were calculated. A *P* value < 0.05 was considered statistically significant.

## Results

During the study period, a total of 11,111 infertile women who underwent fresh embryo transfer met our study criteria (**Figure 1**). Among them, 7,523 patients underwent cleavage-stage embryo transfer, and 3,588 patients underwent blastocyst transfer. We further divided these patients into three groups according to their degree of EMT on the day of hCG trigger. Among patients who underwent cleavage-stage embryo transfer and blastocyst transfer, 115 and 44 patients had an EMT < 7.5 mm, 2,579 and 1,110 patients had an EMT 7.5–12 mm, and 4,829 and 2,434 patients had an EMT ≥ 12 mm, respectively. We defined women with an EMT 7.5–12 mm as the reference group.

**Tables 1**, **2** list the demographic and primary cycle treatment parameter characteristics of the patients. The median EMTs for the groups with EMTs < 7.5 mm, 7.5–12 mm, and ≥ 12 mm were 7.00 mm, 10.00 mm, and 13.00 mm, respectively, in patients who underwent cleavage-stage embryo and blastocyst transfer. As shown in **Tables 1**, **2**, regardless of the type of embryo transferred, the maternal age, gravidity, history of miscarriage, type of infertility, cause of infertility, and E2 levels on the day of hCG trigger significantly differed among the different EMT groups.

**Table 1:** Demographics and baseline characteristics according to different endometrial thickness on trigger day.

Characteristics	Endometrial thickness							
	Patients with cleavage-embryo transfer				Patients with single blastocyst transfer			
	< 7.5 mm (n = 115)	7.5–12 mm (n = 2,579)	≥ 12 mm (n = 4,829)	*P-*value	< 7.5 mm (n = 44)	7.5–12 mm (n = 1,110)	≥ 12 mm (n = 2,434)	*P-*value
Maternal age (years)	34.00 (30.00, 36.00)	31.00 (28.00, 34.00)	30.00 (28.00, 34.00)	<0.001	31.00 (29.00,32.00)	30.00 (27.00,32.00)	29.00 (27.00,32.00)	0.001
Paternal age (years)	33.00 (30.50, 37.00)	32.00 (29.00, 35.00)	31.00 (28.00, 35.00)	<0.001	31.00 (28.75,34.00)	30.00 (28.00,33.00)	30.00 (28.00,33.00)	0.124
Maternal BMI (kg/m^2^)	22.20 (20.40, 24.75)	22.30 (20.40, 24.82)	22.60 (20.50, 24.97)	0.056	23.10 (20.80,26.50)	22.60 (20.52,25.48)	23.10 (20.96,25.60)	0.029
Paternal BMI (kg/m^2^)	25.01 (22.84, 27.28)	25.06 (22.86, 27.44)	25.06 (22.86, 27.44)	0.691	24.01 (21.19,25.99)	24.62 (22.47,27.10)	24.91 (22.80,27.38)	0.007
Infertility duration (years)	2.75 (1.04, 5.00)	3.00 (2.00, 5.00)	3.00 (2.00, 5.00)	0.077	2.79 (2.00,4.00)	3.00 (1.50,4.15)	3.00 (2.00,5.00)	0.010
Gravidity (times)	2.00 (0.00, 3.00)	1.00 (0.00, 2.00)	0.00 (0.00, 1.00)	<0.001	2.00 (0.75, 3.00)	1.00 (0.00, 2.00)	0.00 (0.00, 1.00)	<0.001
Parity (times)	0.00 (0.00, 1.00)	0.00 (0.00, 0.00)	0.00 (0.00, 0.00)	0.015	0.00 (0.00, 0.25)	0.00 (0.00, 0.00)	0.00 (0.00, 0.00)	0.775
History of miscarriage (times)	1.00 (0.00, 2.00)	0.00 (0.00, 1.00)	0.00 (0.00, 0.00)	<0.001	1.00 (0.00, 2.00)	0.00 (0.00, 1.00)	0.00 (0.00, 0.00)	<0.001
Type of infertility, n (%)				<0.001				<0.001
Primary	32 (27.83)	1,142 (44.28)	2,640 (54.67)		11 (25.00)	471 (42.43)	1,255 (51.56)	
Secondary	83 (72.17)	1,437 (55.72)	2,189 (45.33)		33 (75.00)	639 (57.57)	1,179 (48.44)	
Cause of infertility, n (%)				<.001				0.019
Male	7 (6.09)	275 (10.66)	665 (13.77)		3 (6.82)	122 (10.99)	338 (13.89)	
PCOS	9 (7.83)	221 (8.57)	380 (7.87)		7 (15.91)	196 (17.66)	400 (16.43)	
Tubal	48 (41.74)	1,124 (43.58)	1,824 (37.77)		25 (56.82)	471 (42.43)	998 (41.00)	
Uterine	12 (10.43)	57 (2.21)	77 (1.59)		4 (9.09)	39 (3.51)	57 (2.34)	
Cervical	3 (2.61)	16 (0.62)	29 (0.60)		0 (0.00)	8 (0.72)	20 (0.82)	
Endometriosis	3 (2.61)	142 (5.51)	305 (6.32)		0 (0.00)	42 (3.78)	94 (3.86)	
Others	33 (28.70)	744 (28.85)	1,549 (32.08)		5 (11.36)	232 (20.90)	527 (21.65)	
Cycle rank, n (%)				<0.001				0.166
1st	89 (77.39)	2,037 (78.98)	4,019 (83.23)		36 (81.82)	999 (90.00)	2,199 (90.35)	
≥2 cycles	26 (22.61)	542 (21.02)	810 (16.77)		8 (18.18)	111 (10.00)	235 (9.65)	
Maternal education level, n (%)				0.753				0.731
Secondary and below	61 (53.04)	1,399 (54.25)	2,658 (55.04)		26 (59.09)	654 (58.92)	1,468 (60.31)	
Higher	54 (46.96)	1,180 (45.75)	2,171 (44.96)		18 (40.91)	456 (41.08)	966 (39.69)	
Paternal education level, n (%)				0.274				0.430
Secondary and below	56 (48.70)	1,393 (54.01)	2,664 (55.17)		25 (56.82)	657 (59.19)	1,492 (61.30)	
Higher	59 (51.30)	1,186 (45.99)	2,165 (44.83)		19 (43.18)	453 (40.81)	942 (38.70)	
AMH (ng/ml)	2.01 (1.14, 3.03)	2.35 (1.38, 3.76)	2.64 (1.58, 4.07)	<0.001	4.26 (2.60, 5.71)	4.13 (2.80, 5.95)	4.11 (2.84, 5.98)	0.854
AFC	9.00 (6.50, 16.00)	12.00 (7.00, 18.00)	13.00 (9.00, 19.00)	<0.001	16.00 (14.00,22.00)	18.00 (13.00,24.00)	18.00 (14.00,24.00)	0.386
Basal systolic blood pressure (mmHg)	114.00 (107.50, 122.50)	115.00 (108.00, 121.00)	115.00 (108.00, 122.00)	0.666	111.00 (104.75,118.50)	115.00 (108.00,122.00)	115.00 (108.00,122.00)	0.036
Basal diastolic blood pressure (mmHg)	74.00 (69.00, 79.50)	75.00 (70.00, 78.00)	75.00 (69.00, 79.00)	0.512	72.00 (67.75,78.00)	75.00 (70.00,80.00)	76.00 (70.00,80.00)	0.095
Basal FSH (IU/L)	6.69 (5.56, 8.04)	6.63 (5.60, 8.00)	6.61 (5.61, 7.86)	0.664	5.72 (4.97, 6.96)	6.10 (5.22, 7.08)	6.08 (5.22, 7.05)	0.468
COS protocol, n (%)				<0.001				0.141
GnRH agonist	107 (93.04)	2,475 (95.97)	4,703 (97.39)		44 (100.00)	1,088 (98.02)	2,406 (98.85)	
GnRH antagonist	8 (6.96)	104 (4.03)	126 (2.61)		0 (0.00)	22 (1.98)	28 (1.15)	

Continuous variables were expressed as median and interquartile range (IQR) due to non-normally distributed. Categorical variables were expressed as n (%).

Differences were analysed using Kruskal-Wallis rank sum test, Fisher’s exact test or Pearson’s Chi-squared test.

**Table 2:** Cycle parameters according to different endometrial thickness on trigger day.

Characteristics	Endometrial thickness							
	Patients with cleavage-embryo transfer				Patients with single blastocyst transfer			
	< 7.5 mm (n = 115)	7.5–12 mm (n = 2,579)	≥ 12 mm (n = 4,829)	*P-*value	< 7.5 mm (n = 44)	7.5–12 mm (n = 1,110)	≥ 12 mm (n = 2,434)	*P-*value
Total gonadotropin dose (IU)	3,000.00 (1,987.50, 3,618.75)	2,700.00 (1,975.00, 3,450.00)	2,625.00 (1,887.50, 3,375.00)	0.005	1956.25 (1550.00,2768.75)	2037.50 (1500.00,2712.50)	2062.50 (1575.00,2762.50)	0.379
Days of stimulation	12.00 (11.00, 14.00)	13.00 (11.00, 14.00)	13.00 (12.00, 14.00)	0.003	13.00 (11.75, 14.00)	13.00 (12.00, 15.00)	13.00 (12.00, 15.00)	0.086
Endometrial thickness on trigger day (mm)	7.00 (6.00, 7.00)	10.00 (9.00, 11.00)	13.00 (12.00, 15.00)	<0.001	7.00 (7.00, 7.00)	10.00 (9.00, 11.00)	13.00 (12.00, 15.00)	<0.001
E2 level on trigger day (pg/ml)	2,324.00 (1,318.00, 3,657.50)	2,411.00 (1,556.50, 3,497.00)	2,502.00 (1,716.00, 3,595.00)	<0.001	3848.00 (2766.25,5391.75)	3832.00 (2711.00,5385.75)	3531.00 (2498.50,4975.25)	<0.001
Progesterone level on trigger day (ng/ml)	0.84 (0.52, 1.22)	0.81 (0.52, 1.19)	0.82 (0.53, 1.22)	0.478	0.80 (0.60, 1.24)	0.97 (0.66, 1.34)	0.92 (0.61, 1.32)	0.117
Number of oocytes retrieved	9.00 (6.00, 12.00)	10.00 (7.00, 14.00)	11.00 (8.00, 14.00)	<0.001	18.00 (13.00,22.00)	18.00 (14.00,22.00)	18.00 (14.00,21.00)	0.891
Fertilization method, n (%)				0.016				0.220
IVF	90 (78.26)	1,859 (72.08)	3,361 (69.60)		33 (75.00)	853 (76.85)	1,804 (74.12)	
ICSI	25 (21.74)	720 (27.92)	1,468 (30.40)		11 (25.00)	257 (23.15)	630 (25.88)	
Morphological quality of blastocysts transferred, n (%)	/	/	/					0.671
One high quality blastocyst	/	/	/		38 (86.36)	927 (83.51)	2,011 (82.62)	
One poor quality blastocyst	/	/	/		6 (13.64)	183 (16.49)	423 (17.38)	
Morphological quality of cleavage-embryos transferred, n (%)				0.762	/	/	/	/
High quality embryo(s)	104 (90.43)	2,362 (91.59)	4,409 (91.30)		/	/	/	/
One high quality and one low quality embryos	11 (9.57)	200 (7.75)	381 (7.89)		/	/	/	/
Low quality embryo(s)	0 (0.00)	17 (0.66)	39 (0.81)		/	/	/	/
Numbers of cleavage-embryos transfered, n (%)				0.569	/	/	/	/
One	20 (17.39)	358 (13.88)	681 (14.10)		/	/	/	/
Two	95 (82.61)	2,221 (86.12)	4,148 (85.90)		/	/	/	/

Continuous variables were expressed as median and interquartile range (IQR) due to non-normally distributed. Categorical variables were expressed as n (%).

Differences were analysed using Kruskal-Wallis rank sum test, Fisher’s exact test or Pearson’s Chi-squared test.

**Table 3** presents the pregnancy complications and neonatal birth outcomes of singleton live births categorized by EMT group. The overall incidence rates of PTD after cleavage-stage embryo and blastocyst transfer were 6.66% and 8.92%, respectively. In both populations undergoing different types of embryo transfer, the incidence of PTD was greatest in the EMT < 7.5 mm group (15.65% *vs*. 7.64% *vs*. 5.92%, P < 0.001; 27.27% *vs*. 11.26% *vs*. 7.52%, P < 0.001; respectively). In the patients undergoing single blastocyst transfer, the incidence of placenta disorders was greatest in the EMT < 7.5 mm group (6.82% *vs*. 1.53% *vs*. 1.19%, P = 0.021). It suggested that endometrial thickness may be associated with placental abnormalities, thereby influencing neonatal outcomes. Additionally, regardless of the type of embryo transferred, the gestational age at birth and birth weight increased with increasing EMT (*P* < 0.05), whereas the rates of LBW and cesarean section decreased with increasing EMT (*P* < 0.05).

**Table 3:** Pregnancy complications and neonatal outcomes according to different endometrial thickness on trigger day.

Characteristics	Endometrial thickness							
	Patients with cleavage-embryo transfer				Patients with single blastocyst transfer			
	< 7.5 mm (n = 115)	7.5–12 mm (n = 2,579)	≥ 12 mm (n = 4,829)	*P-*value	< 7.5 mm (n = 44)	7.5–12 mm (n = 1,110)	≥ 12 mm (n = 2,434)	*P-*value
Placenta disorders, n (%)				0.171				0.021
No	112 (97.39)	2,539 (98.45)	4,771 (98.80)		41 (93.18)	1,093 (98.47)	2,405 (98.81)	
Yes	3 (2.61)	40 (1.55)	58 (1.20)		3 (6.82)	17 (1.53)	29 (1.19)	
PROM, n (%)				0.187				0.011
No	110 (95.65)	2,510 (97.32)	4,720 (97.74)		40 (90.91)	1,068 (96.22)	2,372 (97.45)	
Yes	5 (4.35)	69 (2.68)	109 (2.26)		4 (9.09)	42 (3.78)	62 (2.55)	
GDM, n (%)				0.076				0.040
No	104 (90.43)	2,426 (94.07)	4,576 (94.76)		42 (95.45)	1,031 (92.88)	2,312 (94.99)	
Yes	11 (9.57)	153 (5.93)	253 (5.24)		2 (4.55)	79 (7.12)	122 (5.01)	
HDP, n (%)				0.692				0.549
No	111 (96.52)	2,504 (97.09)	4,698 (97.29)		42 (95.45)	1,081 (97.39)	2,371 (97.41)	
Yes	4 (3.48)	75 (2.91)	131 (2.71)		2 (4.55)	29 (2.61)	63 (2.59)	
Preterm delivery, n (%)				<0.001				<0.001
No	97 (84.35)	2,382 (92.36)	4,543 (94.08)		32 (72.73)	985 (88.74)	2,251 (92.48)	
Yes	18 (15.65)	197 (7.64)	286 (5.92)		12 (27.27)	125 (11.26)	183 (7.52)	
Gender of newborn, n (%)				0.044				0.158
Female	53 (46.09)	1,310 (50.79)	2,310 (47.84)		24 (54.55)	464 (41.80)	1,070 (43.96)	
Male	62 (53.91)	1,269 (49.21)	2,519 (52.16)		20 (45.45)	646 (58.20)	1,364 (56.04)	
Gestational age (weeks)	38.00 (37.00, 39.00)	39.00 (38.00, 39.00)	39.00 (38.00, 40.00)	<0.001	38.00 (36.00, 39.00)	38.00 (38.00, 39.00)	39.00 (38.00, 39.00)	<0.001
Birth weight (g)	3,300 (3,000, 3,600)	3,300 (3,050, 3,645)	3,400 (3,100, 3,700)	<0.001	3,150 (2,748, 3,325)	3,350 (3,050, 3,650)	3,400 (3,100, 3,700)	<0.001
LBW, n (%)				<0.001				0.003
No	101 (87.83)	2,475 (95.97)	4,649 (96.27)		38 (86.36)	1,063 (95.77)	2,356 (96.80)	
Yes	14 (12.17)	104 (4.03)	180 (3.73)		6 (13.64)	47 (4.23)	78 (3.20)	
Macrosomia, n (%)				0.093				0.001
No	110 (95.65)	2,407 (93.33)	4,452 (92.19)		44 (100.00)	1,035 (93.24)	2,198 (90.30)	
Yes	5 (4.35)	172 (6.67)	377 (7.81)		0 (0.00)	75 (6.76)	236 (9.70)	
SGA, n (%)				0.950				0.888
No	109 (94.78)	2,441 (94.65)	4,579 (94.82)		42 (95.45)	1,067 (96.13)	2,340 (96.14)	
Yes	6 (5.22)	138 (5.35)	250 (5.18)		2 (4.55)	43 (3.87)	94 (3.86)	
LGA, n (%)				0.113				0.046
No	87 (75.65)	2,009 (77.90)	3,658 (75.75)		36 (81.82)	835 (75.23)	1,749 (71.86)	
Yes	28 (24.35)	570 (22.10)	1,171 (24.25)		8 (18.18)	275 (24.77)	685 (28.14)	
Delivery mode, n (%)				<0.001				0.021
Vaginal	23 (20.00)	679 (26.33)	1,473 (30.50)		8 (18.18)	291 (26.22)	730 (29.99)	
Caesarean section	92 (80.00)	1,900 (73.67)	3,356 (69.50)		36 (81.82)	819 (73.78)	1,704 (70.01)	

Continuous variables were expressed as median and interquartile range (IQR) due to non-normally distributed. Categorical variables were expressed as n (%).

Differences were analysed using Kruskal-Wallis rank sum test, Fisher’s exact test or Pearson’s Chi-squared test.

We further employed logistic regression models to assess the correlation between EMT on the day of the hCG trigger and PTD. We present the results as ORs for the crude model and two different adjusted models (**Table 4**). Compared with the population with an EMT of 7.5–12 mm, the population with an EMT < 7.5 mm had a 2.24-fold (OR 2.24; 95% CI, 1.33–3.79; *P* = 0.002) and 2.96-fold (OR 2.96; 95% CI, 1.48–5.89; *P* = 0.002) increased probability of PTD in populations undergoing cleavage-stage embryo transfer and blastocyst transfer, respectively, in the crude model, which was not adjusted for any variables. Moreover, the population with an EMT ≥ 12 mm had a 24% (OR 0.76; 95% CI, 0.63–0.92; *P* = 0.004) and 36% (OR 0.64; 95% CI, 0.50–0.81; *P* < 0.001) reduced risk of PTD, respectively, using the same model. In Model I, which was partially adjusted based on the baseline characteristics and cycle treatment parameters, the risk of PTD was increased by 1.88-fold (OR 1.88; 95% CI, 1.10–3.24; *P* = 0.022) and 2.89-fold (OR 2.89; 95% CI, 1.44–5.82; *P* = 0.003) in the EMT < 7.5 mm group compared with the EMT 7.5–12 mm group, whereas the risk of PTD was reduced by 23% (OR 0.77; 95% CI, 0.63–0.93; *P* = 0.007) and 37% (OR 0.63; 95% CI, 0.50–0.81; *P* < 0.001) in the EMT ≥ 12 mm group. In the fully adjusted model (Model II), we also considered maternal pregnancy complications and fetal intrauterine development. This model was adjusted for the variables in Model I as well as placental abnormalities, PROM, GDM, HDP, gender of the newborn, SGA, and LGA. We found that even after adjusting for all variables, an EMT ≥ 12 mm retained its independent protective effect against PTD in both cleavage-stage embryo and blastocyst transfer populations (OR 0.74; 95% CI, 0.58–0.95; *P* = 0.018; *vs*. OR 0.62; 95% CI, 0.46–0.85; *P* = 0.003, respectively). In contrast, in the blastocyst transfer population, no significant correlation was detected between EMT < 7.5 mm and PTD (OR 2.19; 95% CI, 0.82–5.88; *P* = 0.118), whereas EMT < 7.5 mm remained an independent risk factor for PTD in the cleavage embryo transfer population (OR 2.14; 95% CI, 1.09–4.21; *P* = 0.027).

**Table 4:** Association between endometrial thickness and PTD in different models.

Patients	Variables	Crude Model ^a^		Adjusted Model I ^b^		Adjusted Model II ^c^	
		OR (95% CI)	*P-*value	OR (95% CI)	*P-*value	OR (95% CI)	*P-*value
Patients with cleavage-embryo transfer	**PTD**						
	**EMT**						
	**7.5–12 mm**	**Reference**		**Reference**		**Reference**	
	**<7.5 mm**	2.24(1.33, 3.79)	0.002**	1.88(1.10, 3.24)	0.022*	2.14(1.09, 4.21)	0.027*
	**≥12 mm**	0.76(0.63, 0.92)	0.004**	0.77(0.63, 0.93)	0.007**	0.74(0.58, 0.95)	0.018*
Patients with single blastocyst transfer	**PTD**						
	**EMT**						
	**7.5–12 mm**	**Reference**		**Reference**		**Reference**	
	**<7.5 mm**	2.96 (1.48, 5.89)	0.002^**^	2.89 (1.44, 5.82)	0.003^**^	2.19 (0.82, 5.88)	0.118
	**≥12 mm**	0.64 (0.50, 0.81)	<0.001^***^	0.63 (0.50, 0.81)	<0.001^***^	0.62 (0.46, 0.85)	0.003^**^

OR, Odds Ratio; CI, Confidence Interval; *p<0.05; **p<0.01; ***p<0.001.

a Crude model was not adjusted for other covariants.

b Model I was adjusted for the baseline characteristics and the cycle parameters: maternal age, paternal age, maternal BMI, paternal BMI, infertility duration, gravidity, parity, history of miscarriage, type of infertility, cause of infertility (including male, PCOS, tubal, uterine, cervical, endometriosis, others), cycle rank, maternal education level, paternal education level, AMH, AFC, basal systolic blood pressure, basal diastolic blood pressure, basal FSH, COS protocol, total gonadotropin dose, days of stimulation, E2 level on trigger day, progesterone level on trigger day, number of oocytes retrieved, fertilization method, number of embryos transfered, morphological quality of embryos transferred.

c Model II was adjusted for all the covariants in Model I plus the pregnancy complications index: placenta disorder, PROM, GDM, HDP, gender of newborn, SGA, LGA.

## Discussion

The main objective of our study was to evaluate the relationship between EMT on the day of trigger and PTD in a population of singleton live births undergoing fresh IVF/ICSI cycles. In this single-center retrospective cohort study, which included 11,111 singleton live births from infertile women aged ≤ 42 years, we found a strong correlation between EMT and PTD. After adjusting for potential confounders, such as the demographic characteristics of the couples and the cycle treatment parameters, women with an EMT < 7.5 mm had a greater risk of PTD. After confounding factors, such as maternal complications during pregnancy were considered, an EMT < 7.5 mm was an independent risk factor for PTD in the population undergoing cleavage-stage embryo transfer. In contrast, in the population that underwent blastocyst transfer, an EMT < 7.5 mm was no longer an independent risk factor for PTD, whereas an EMT ≥ 12 mm still maintained a negative correlation with PTD. These data suggest that EMT on the day of hCG trigger may be associated with the occurrence of PTD in the population undergoing fresh embryo transfer. Regardless of the type of transfer (cleavage-stage or blastocyst transfer), a thicker the endometrium suggested a lower risk of PTD. Especially in women with potential pregnancy complications, blastocyst transfer combined with a sufficiently thick endometrium may reduce the risk of PTD. Therefore, we suggest blastocyst transfer in patients with thin endometrium to optimize pregnancy outcomes. This clinical decision may be more cost-effective by reducing the incidence of PTD and other complications, thereby lowering medical expenses.

The relationship between EMT and pregnancy outcomes in ART has long been a focus of researchers (8, 28). A recent meta-analysis revealed that a thin endometrium is associated with a high risk of LBW, SGA, placental abruption, HDP, cesarean section and PTD (29). In populations with polycystic ovary syndrome (PCOS), reduced EMT is independently associated with increased risks of PTD, LBW, and SGA (30). In a study of frozen cleavage-stage embryo transfer, the PTD incidence was 9.4% (15), which is similar to our findings. Moreover, patients with an EMT < 8 mm had a significantly greater risk of PTD and LBW (*P* < 0.05) in the same study. A recent study involving 2,010 singleton deliveries confirmed that in fresh cycles, compared with an EMT of 6.00–8.90 mm, the likelihood of PTD was reduced by 75% when the EMT was greater than 11 mm (aOR 0.25; 95% CI, 0.04–0.66; *P* = 0.0034), and smooth curve fitting and threshold effect analysis identified 7.8 mm as the cutoff value for predicting PTD (14). However, that study did not differentiate between cleavage and blastocyst transfers and excluded populations with pregnancy complications.

Previous studies on the association between EMT and PTD in fresh cycles did not distinguish between the types of embryo transferred, which may have led to confounding results. Therefore, we chose to conduct the study separately in populations undergoing cleavage-stage embryo transfer and blastocyst transfer. Consistent with previous results, we found that when maternal pregnancy complications were not considered, an EMT < 7.5 mm was an independent risk factor for PTD and an EMT ≥ 12 mm was an independent protective factor, regardless of the type of embryo transferred. When we extended the follow-up period and included maternal pregnancy complications, we found that a thin endometrium remained an independent risk factor for PTD in cleavage-stage embryo transfer; however, in the blastocyst transfer population, the positive correlation between a thin endometrium and PTD became statistically insignificant, suggesting that maternal pregnancy complications may have had a greater impact on PTD at that time. The differences between cleavage-stage embryos and blastocyst transfers are not only reflected in the association between EMT and PTD. Recent studies have also reported differences. For example, a meta-analysis conducted in 2022 revealed that in fresh cycles, the live birth rate following blastocyst transfer was greater than that after cleavage-stage embryo transfer, but it increased the risk of obstetric and perinatal complications by 2–3% (31). In singleton pregnancies from fresh IVF cycles, patients who underwent blastocyst transfer had a higher risk of PP (aOR 2.11; 95% CI, 1.76-2.52) as well as a higher risk of PTD (aOR 1.14; 95% CI, 1.01; 1.29) (32). The results of two recent high-quality evidence multicenter randomized controlled trials published in 2024 reaffirmed this conclusion (33, 34). These differences could be explained by the suboptimal conditions of extended *in vitro* culture compared with *in vivo* culture for implantation and placentation. Additionally, this difference may also be related to the different cross-talks between embryos of varying *in vitro* culture durations and the endometrium. The implantation process depends not only on the “readiness” of the endometrium but also on the complex interactions between the endometrium and embryo, which communicate through various molecules (growth factors, cytokines, and active factors) via crosstalk. Some studies have shown that the level of vascular endothelial growth factor (VEGF) in the culture medium during the blastocyst stage is significantly greater than that in early-stage embryos (35). Therefore, we speculate that certain growth factors, cytokines, and active factors released by blastocyst-stage embryos might compensate for some of the negative impacts on outcomes due to insufficient endometrial development. However, regardless of the type of embryo transferred, an EMT ≥ 12 mm retained its protective effect against PTD. The underlying mechanism may involve the thick endometrium can improve the blood flow and nutrient supply, thereby promote the placental development and fetal growth.

Currently, the mechanisms by which EMT has effects on embryo implantation, placental development, and offspring safety remain under investigation. First, this process may be related to the vascular development of the endometrium. Normally, a low-resistance blood flow connection is established between the spiral arteries and the uterine arteries, which increases the circulating blood flow in the intervillous space and the placenta and provides sufficient nutrients for fetal growth and development (36). Defects in spiral artery remodeling may be one of the reasons for a spectrum of obstetrical syndromes (37). In fact, an important characteristic of a thin endometrium is the increased resistance of the uterine arteries, which affects the normal blood supply to the placenta (38). In endometrial tissue samples suspected of having defects, researchers have reported insufficient or completely absent expression of leukemia inhibitory factor (LIF), VEGF, and β3 integrin (39). Severe deficiency of these angiogenic factors may be the primary cause of vascular abnormalities in thin endometria. Second, the negative effects of a thin endometrium on neonatal outcomes may also be related to poor decidualization. The endometrium decidualizes after pregnancy and develops into a part of the placenta. A recent study involving single-cell RNA sequencing of human endometrial cells revealed that in thinner endometria, cell cycle signaling pathways are impaired, collagen deposition around blood vessels is excessive, and the number of macrophages and natural killer cells is reduced, all of which lead to the poor decidualization of a thin endometrium (40). Poor decidualization and local microenvironment abnormalities in thin endometria may further result in spiral artery remodeling. Another possible mechanism is the difference in oxygen tension between thin endometrium and normal endometrium. When the functional layer of the endometrium is thin or absent, the oxygen concentration in the basal endometrial blood vessels increases significantly. In patients with a thinner endometrium, the placenta and fetus may be closer to the basal endometrium. This area has richer blood flow and higher oxygen content, which may lead to impaired fetal growth (41). Therefore, more free radicals may be produced, potentially leading to impaired fetal growth.

We believe that our research is a valuable supplement to existing scholarly works. First, we included only fresh embryo transfer cycles and distinguished between different types of embryos, which minimized the confounding effects of these factors on the outcomes. Second, based on previous studies, we selected 7.5 mm and 12 mm as the lower and upper cutoff values for EMT, respectively, which is consistent with conventional methods. Moreover, we adjusted for a more comprehensive set of confounding factors, including baseline blood pressure and data on maternal pregnancy complications, which increased the robustness of our results. However, some limitations are worth noting. First, the retrospective nature of the study limits the control of confounding factors. Second, the current study did not consider maternal lifestyle characteristics, including alcohol consumption, smoking, diet, and exercise habits. Furthermore, this study was conducted in a single ART center, and the characteristics of the study population may limit the generalizability of the findings. Future large-sample multicenter randomized controlled trials are needed to address these limitations and provide more robust evidence.

## Conclusions

In summary, our study indicates that in patients undergoing fresh cleavage-stage embryo transfer, a thin endometrium (EMT < 7.5 mm) on the day of hCG trigger is independently associated with an increased risk of PTD. However, in patients undergoing blastocyst transfer, a thin endometrium is not independently associated with the occurrence of PTD. Conversely, a thick endometrium (EMT ≥ 12 mm) is independently associated with a decreased risk of PTD in both populations. These results underscore the importance of monitoring the EMT during COS treatment to optimize neonatal health outcomes. Therefore, to reduce the risk of PTD, we recommend that infertile women with potential pregnancy complications should be selected for blastocyst transfer when the endometrium is sufficiently thick.

## Data Availability

The raw data supporting the conclusions of this article will be made available by the authors, without undue reservation.
